# MicroRNA-125b-1-3p mediates intervertebral disc degeneration in rats by targeting teashirt zinc finger homeobox 3

**DOI:** 10.3892/etm.2021.10935

**Published:** 2021-10-28

**Authors:** Xiaotong Meng, Yue Zhu, Lin Tao, Sichao Zhao, Shui Qiu

Exp Ther Med 13:2627–2633, 2018; DOI: 10.3892/etm.2018.5715

After the publication of the above article, the authors have realized that the β-actin control bands were selected incorrectly for Figs. 2A and 5A. These errors arose inadvertently as a consequence of the authors’ misfiling of their data.

The revised versions of Figs. 2 and 5, featuring the correct β-actin control bands for Figs. 2A and 5A, are shown opposite. Note that the revised data shown for these Figures do not affect the overall conclusions reported in the paper. The authors are grateful to the Editor of *Experimental and Therapeutic Medicine* for allowing them the opportunity to publish ths Corrigendum, and to the readership for any inconvenience caused.

## Figures and Tables

**Figure 2:**
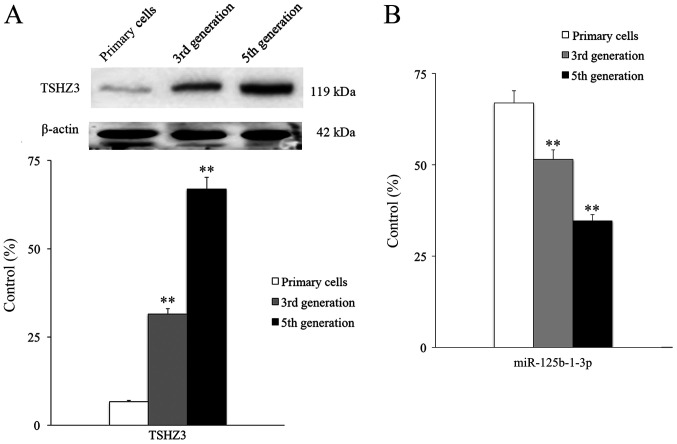
Expression of TSHZ3 and miR-125b-1-3p in NP cells. (A) The expression of TSHZ3 in NP cells from different generations. (B) The expression of miR-125b-1-3p in NP cells from different generations. ^**^P<0.01 vs. primary cells. NP, nucleus pulposus; TSHZ3, Teashirt zinc finger homeobox 3; miR, microRNA.

**Figure 5:**
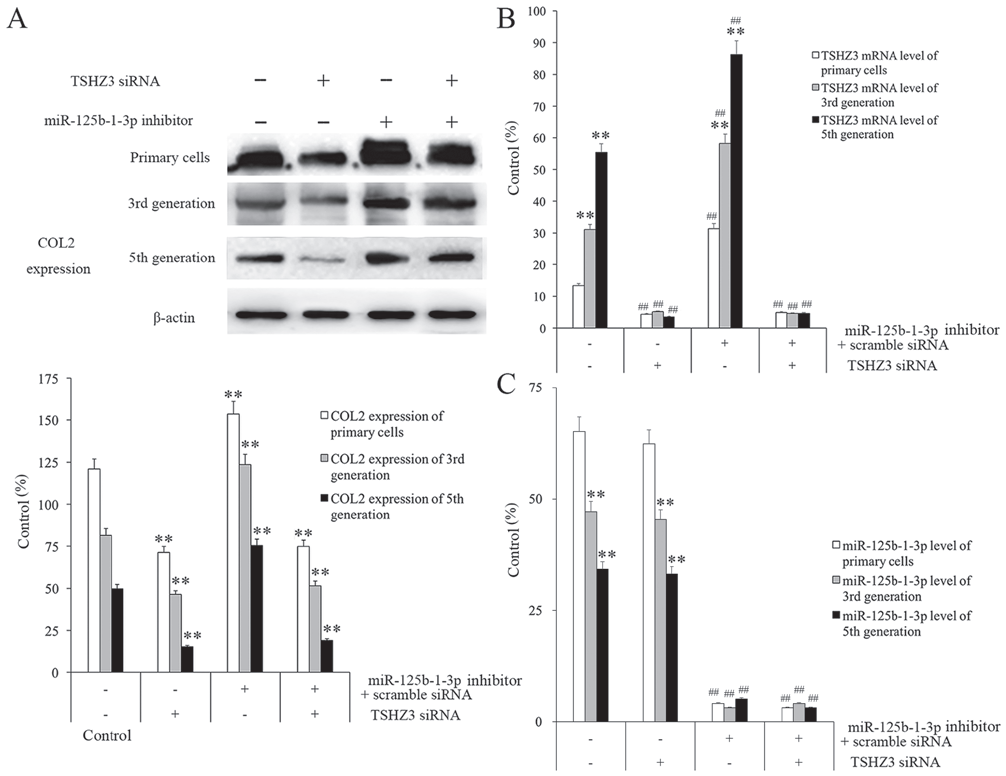
The effect of transfection with miR-125b-1-3p inhibitor and TSHZ3 siRNA on NP cells. TSHZ3 siRNA alone or combined with miR-125b-1-3p inhibitor was administered to NP cells and the expression of (A) COL2, (B) TSHZ3 and (C) miR-125b-1‐-p was measured from different generations of NP cells. ^**^P<0.01 vs. control cells among the generations. ^##^P<0.01 vs. the control cells (inhibitor- and TSHZ3 siRNA-) among the treated groups in (B) and (C). miR, microRNA. NP, nucleus pulposus; TSHZ3, Teashirt zinc finger homeobox 3; miR, microRNA; COL2, collagenase type II; siRNA, small interfering RNA.

